# Absence of chest discomfort in type 1 NSTEMI patients: predictors and impact on outcome

**DOI:** 10.1007/s00392-025-02628-1

**Published:** 2025-03-13

**Authors:** J. Michael Altstidl, Merve Günes-Altan, Maximilian Moshage, Florian Weidinger, Lennart Lorenz, Dominik Weimann, Christina Chapuzot, Monique Tröbs, Mohamed Marwan, Stephan Achenbach, Luise Gaede

**Affiliations:** Department of Medicine 2 – Cardiology and Angiology, Friedrich-Alexander-Universität Erlangen-Nürnberg, Ulmenweg 18, 91054 Erlangen, Germany

**Keywords:** Non-ST-elevation myocardial infarction, Symptoms, Disparities, In-hospital outcomes, Registry

## Abstract

**Background:**

The absence of chest discomfort has been hypothesized to delay treatment and consequently result in worse outcomes in patients with non-ST-elevation myocardial infarction (NSTEMI).

**Methods:**

In 888 consecutive patients with type 1 NSTEMI, symptoms were systematically classified as chest discomfort defined as chest pain or pressure, dyspnea or other symptoms, e.g. epigastric pain. Patient characteristics predictive for the absence of chest discomfort and the impact of the symptom type on adverse in-hospital events (all-cause mortality, cardiogenic shock, and mechanical ventilation) were analyzed.

**Results:**

Chest discomfort was reported in 81.0%, dyspnea without chest discomfort in 12.2%, and only other symptoms in the remaining 6.9% of patients. In a multivariable regression analysis, female sex (*p* = 0.035), diabetes mellitus (*p* = 0.003), the absence of any family history of coronary artery disease (CAD) (*p* = 0.002), anemia (*p* < 0.001), and atrial fibrillation or flutter at presentation (*p* = 0.017) were independent predictors for the absence of chest discomfort.

The absence of chest discomfort was associated with a higher rate of in-hospital adverse events (10.6% for chest discomfort vs. 29.6% for dyspnea and 27.9% for other symptoms, *p* < 0.001), which appeared partially mediated (*p* = 0.044) by longer times from diagnosis to invasive management (*p* < 0.001).

**Conclusions:**

In type 1 NSTEMI, the absence of chest discomfort is associated with a higher rate of adverse in-hospital events. Women, diabetics, patients without a family history of CAD, patients with anemia, and patients with atrial fibrillation are more likely to present without chest discomfort and special attention may be required to avoid delayed invasive management in these patients.

**Graphical Abstract:**

**Figure:**
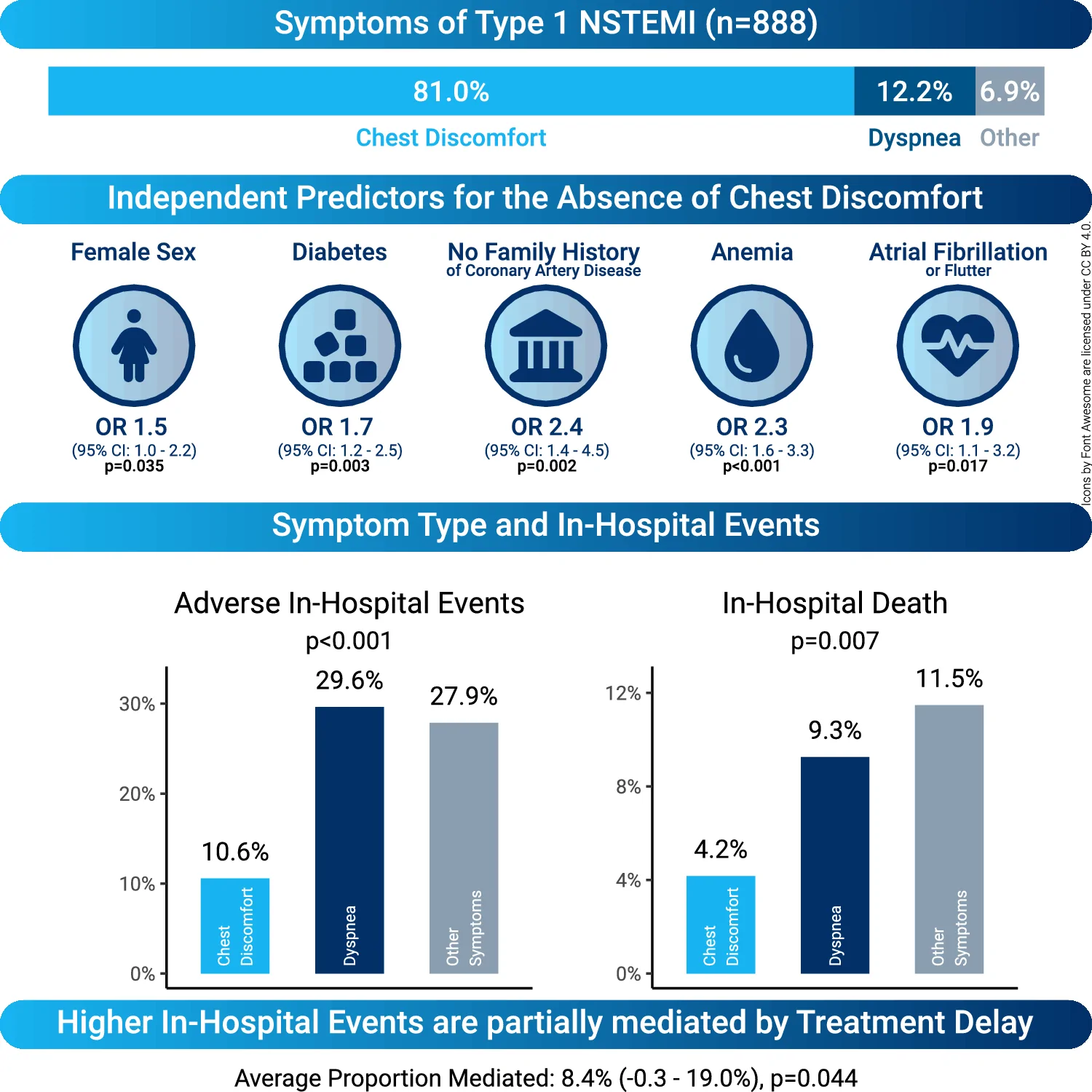

**Supplementary Information:**

The online version contains supplementary material available at https://doi.org/10.1007/s00392-025-02628-1.

## Introduction

Most patients with acute coronary syndromes present with typical symptoms, defined as chest pain or pressure [1], but others have less obvious symptoms [1]. However, early diagnosis is crucial for timely treatment. This can be especially challenging when no evidence of ischemia can be found on the electrocardiogram, as is often the case in non-ST-segment elevation myocardial infarction (NSTEMI).

Since shorter times to revascularization have been clearly demonstrated to improve survival in patients with ST-segment elevation myocardial infarction (STEMI) [2–4], immediate invasive angiography and percutaneous coronary intervention (PCI) as needed is recommended by current guidelines [1, 5]. Given the emerging evidence for the benefit of an early intervention also in patients with NSTEMI [6, 7], the ESC guidelines now equally recommend an immediate invasive strategy in selected patients with NSTEMI presumed to be at very high risk [1]. However, some patients with NSTEMI present with atypical symptoms—according to prior data, especially women [8] and older patients [9, 10]. This might lead to undertreatment or treatment delays of patients which in turn may cause unfavorable outcome [11–14].

Therefore, this study aims to analyze the presenting symptoms of patients with NSTEMI, and their variation caused by demographics, comorbidities, and culprit lesion location as well as to investigate the association between presenting symptom characteristics on in-hospital outcome in a large, single-center, all-comers, real-world registry.

## Methods

### Study design

Consecutive patients transferred to the cardiac catheterization laboratory due to a suspected NSTEMI based on the currently recommended ESC troponin algorithms [1] were prospectively included in a single-center, all-comers, real-world registry between February 2018 and December 2023.

**Fig. 1:**
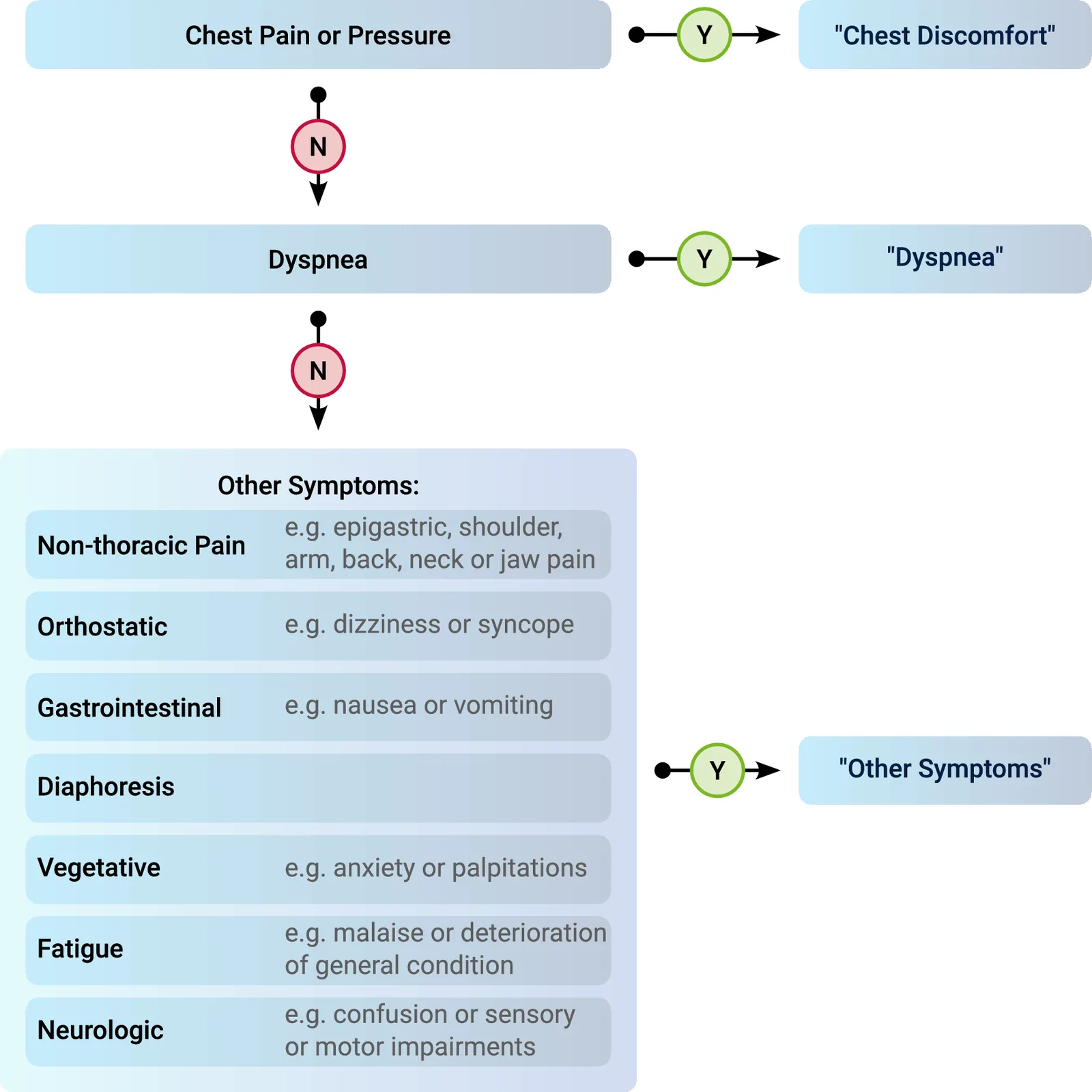
Categorization of symptoms. Flowchart for categorization of patients regarding the primary symptom as “chest discomfort”, “dyspnea”, or “other symptoms” depending on the presenting symptoms

Symptoms were obtained as part of the routine initial workup. Consistent with the current ESC guidelines, symptoms were classified as chest pain or pressure (pain, pressure, tightness, heaviness, or burning), dyspnea (shortness of breath), non-thoracic pain (e.g. epigastric, shoulder, arm, back, neck or jaw pain), orthostatic symptoms (e.g. dizziness or syncope), gastrointestinal symptoms (e.g. nausea or vomiting), diaphoresis, other vegetative symptoms (e.g. anxiety or palpitations), fatigue (e.g. malaise or deterioration of general condition), and neurologic symptoms (e.g. confusion or sensory or motor impairments) [1]. Patients were categorized into three groups depending on the primary symptom as shown in Fig. 1: If chest pain or pressure was present, the patient was categorized into the group “chest discomfort”. If no chest pain or pressure but dyspnea was present, the patient was categorized into the group “dyspnea”. If neither chest pain or pressure nor dyspnea was present, the patient was categorized into the group “other symptoms”.

Based on angiographic findings, patients were divided into type 1 and type 2 NSTEMI. To clarify that only NSTEMI patients are included, the terms “type 1 NSTEMI” and “type 2 NSTEMI” are used, which are adapted from the definitions “type 1 myocardial infarction” and “type 2 myocardial infarction” of the fourth universal definition of myocardial infarction [15]. Patients were categorized to type 1 NSTEMI if a culprit lesion causing the acute myocardial ischemia could be identified, while patients were categorized to type 2 NSTEMI when no culprit lesion was evident. Only patients with type 1 NSTEMI were included in the analysis. For the analysis of the influence of age on symptoms, patients ≥ 80 years were classified as elderly and patients with a body mass index ≥ 30 kg/m^2^ were classified as obese. A positive family history of premature coronary artery disease (CAD) was defined as 1 or more first-degree relatives with early signs of CAD, i.e. before 55 years of age in men and before 65 years of age in women [16]. For all analyses, patients were excluded if the analyzed variable was not obtained or could not be determined.

Furthermore, the influence of the primary symptom on in-hospital death, cardiogenic shock, need of mechanical ventilation, the development of acute kidney injury, the thrombolysis in myocardial infarction (TIMI) flow of the culprit lesion after PCI, the rate of no reflow phenomenon, the left ventricular ejection fraction as determined by echocardiography, and the peak of the cardiac biomarkers troponin and creatine kinase were analyzed. Adverse in-hospital events comprised the composite of in-hospital death, cardiogenic shock, and mechanical ventilation. Acute kidney injury was defined by an increase in serum creatinine based on the Acute Kidney Injury Network (AKIN) classification [17]. Anemia was defined as a hemoglobin concentration < 12 g/dL in women and < 13 g/dL in men [18].

Finally, the mediating effect of the time from diagnosis to invasive management on the influence of the primary symptom on adverse in-hospital events was analyzed. The time of diagnosis was based on when the diagnosis was clinically established considering the combination of clinical presentation, electrocardiogram and troponin in accordance with the ESC guidelines [1]. The time of invasive management was defined by the moment of the first subcutaneous injection of local anesthesia for invasive coronary angiography in the catheterization laboratory. This study was performed in accordance with the ethical standards as laid down in the 1964 Declaration of Helsinki and its later amendments. The study protocol was approved by the institutional ethics committee.

### Statistical analysis

Statistical analysis was performed with R and its *table1*, *tidyverse*, *gtsummary* and *mediation* libraries [19–23]. Continuous variables are expressed as medians and interquartile ranges (IQR), while categorial characteristics are expressed as absolute values and percentages. Data were compared with the chi-squared test for categorical variables and with the Wilcoxon rank sum test for two groups or the Kruskal–Wallis test for three or more groups for continuous variables. Relevant independent predictors of chest discomfort were determined by a multivariable regression analysis only including potential predictors significant in a univariable regression analysis. The odds ratio (OR) and 95% confidence interval (CI) are given for each variable of the regression analysis. To investigate mediating effects, causal mediation analysis was performed. For all analyses, a *p* value < 0.05 was considered to indicate statistical significance.

## Results

Of the 1192 patients transferred to the cardiac catheterization laboratory due to a diagnosed NSTEMI between February 2018 and December 2023, a type 1 NSTEMI could be identified in 950 patients. The documentation of symptoms was not complete in 62 of these patients, who were therefore excluded from the analysis. Of these patients, 29 were resuscitated beforehand. Other reasons for the lack of an explicit symptom history were, for example, dementia or language barriers.

Patient characteristics are described in Table 1 and symptoms are outlined in Table 2. Chest discomfort was reported by 81.0%. Dyspnea was reported by 43.6% with it being the primary symptom in 12.2%. Other symptoms were reported by 39.0% with it being the primary symptom in 6.9%. More specifically, non-thoracic pain was reported by 20.8%, orthostatic symptoms by 24.9%, gastrointestinal symptoms by 20.8%, diaphoresis by 19.1%, other vegetative symptoms by 16.5%, fatigue by 12.4% and neurologic symptoms by 7.2%.

**Table 1:** Patient characteristics (*n* = 888 patients)

Age, years	69 [60–79]
Sex, male	662 (74.5%)
Obesity*	193 (21.7%)
Hypertension	736 (82.9%)
Diabetes mellitus	288 (32.4%)
Dyslipidemia	658 (74.1%)
Smoking	357 (40.2%)
Family history of CAD	181 (20.4%)
Previous PCI	284 (32.0%)
Previous CABG	78 (8.8%)
Creatinine, mg/dl	0.99 [0.83–1.24]
Hemoglobin, mg/dl	13.7 [11.9–14.9]
Troponin, pg/ml	5140 [1500–19000]
Creatine kinase, U/l	366 [186–812]
Hearth rhythm on ECG	
Sinus rhythm	806 (90.8%)
Atrial fibrillation/flutter	80 (9.0%)
Other	2 (0.2%)
Ischemic ECG changes	416 (46.8%)
Ejection fraction*, %	45 [40–55]
Culprit lesion	
LAD	339 (38.2%)
LCX	221 (24.9%)
RCA	208 (23.4%)
LM	26 (2.9%)
Graft	23 (2.6%)
Multiple	71 (8.0%)
None	–

Values are median [IQR] or *n* (%)

*CABG* indicates coronary artery bypass graft, *CAD* coronary artery disease, *LAD* left anterior descending artery, *LCX* left circumflex artery, *LM* left main coronary artery, *PCI* percutaneous coronary intervention, *RCA* right coronary artery

^*^Body mass index missing for 131 (11.9%) patients and ejection fraction for 22 (2.0%) patients

**Table 2:** Symptoms (*n* = 888 patients)

Chest discomfort	719 (81.0%)
Dyspnea	387 (43.6%)
Other symptoms	346 (39.0%)
Non-thoracic pain	72/346 (20.8%)
Orthostatic	86/346 (24.9%)
Gastrointestinal	72/346 (20.8%)
Diaphoresis	66/346 (19.1%)
Vegetative	57/346 (16.5%)
Fatigue	43/346 (12.4%)
Neurologic	25/346 (7.2%)
Primary symptom	
Chest discomfort	719 (81.0%)
Dyspnea	108 (12.2%)
Other symptoms	61 (6.9%)

Values are *n* (%)

The exact time of the onset of symptoms was recalled only by 30.5% of patients. Patients with chest discomfort were more likely to remember the exact time of symptom onset (34.9% with chest discomfort, 7.4% with dyspnea, and 19.7% with other symptoms, *p* < 0.001).

### Patient characteristics by primary symptom in type 1 NSTEMI

Patients with type 1 NSTEMI presenting with chest discomfort (68 [IQR 59–78] years) were significantly younger than patients who reported dyspnea (74 [IQR 66–83] years) or other symptoms (71 [IQR 63–79] years, *p* < 0.001). The proportion of women was lowest for chest discomfort (23.8%) and highest for other symptoms (39.3%, *p* = 0.020). Diabetes mellitus was most frequently present in patients with dyspnea (48.1%) and least frequent in patients with chest discomfort (29.6%, *p* < 0.001), whereas a family history of premature CAD was most frequent in patients with chest discomfort (23.1%) and lowest among patients with dyspnea (7.4%, *p* < 0.001). A history of smoking was most prevalent in patients with chest discomfort (42.0%) and least prevalent in patients with other symptoms (26.2%, *p* = 0.036). Creatinine values at presentation were highest for patients presenting with dyspnea (1.05 mg/dl [IQR 0.85–1.52 mg/dl]) and lowest for those presenting with chest discomfort (0.97 mg/dl [IQR 0.83–1.21 mg/dl], *p* = 0.015). Hemoglobin concentration at presentation was lowest for patients presenting with dyspnea (12.0 mg/dl [IQR 10.2–13.5 mg/dl]) and highest for those presenting with chest discomfort (13.9 mg/dl [IQR 12.3–15.1 mg/dl], *p* < 0.001). Atrial fibrillation or flutter was most frequent in patients with dyspnea (16.7%) and least frequent in patients with chest discomfort (7.2%, *p* < 0.001). Patient characteristics stratified by the primary symptom for patients with type 1 NSTEMI are outlined in detail in Table 3.

**Table 3:** Patient characteristics by primary symptom (*n* = 888 patients)

	Chest discomfort (*n* = 719)	Dyspnea (*n* = 108)	Other symptoms (*n* = 61)	*P* value
Age, years	68 [59–78]	74 [66–83]	71 [63–79]	** < 0.001**
Sex, male	548 (76.2%)	77 (71.3%)	37 (60.7%)	**0.020**
Obesity*	163 (22.7%)	19 (17.6%)	11 (18.0%)	0.584
Hypertension	596 (82.9%)	94 (87.0%)	46 (75.4%)	0.156
Diabetes mellitus	213 (29.6%)	52 (48.1%)	23 (37.7%)	** < 0.001**
Dyslipidemia	539 (75.0%)	79 (73.1%)	40 (65.6%)	0.267
Smoking	302 (42.0%)	39 (36.1%)	16 (26.2%)	**0.036**
Family history of CAD	166 (23.1%)	8 (7.4%)	7 (11.5%)	** < 0.001**
Previous PCI	231 (32.1%)	36 (33.3%)	17 (27.9%)	0.751
Previous CABG	66 (9.2%)	7 (6.5%)	5 (8.2%)	0.644
Creatinine, mg/dl	0.97 [0.83–1.21]	1.05 [0.85–1.52]	1.04 [0.82–1.25]	**0.015**
Hemoglobin, mg/dl	13.9 [12.3–15.1]	12.0 [10.2–13.5]	13.1 [10.7–14.4]	** < 0.001**
Hearth rhythm on ECG				
Sinus rhythm	667 (92.8%)	89 (82.4%)	50 (82.0%)	** < 0.001**
Atrial fibrillation/flutter	52 (7.2%)	18 (16.7%)	10 (16.4%)	
Other	0 (0%)	1 (0.9%)	1 (1.6%)	
Culprit lesion				
LAD	274 (38.1%)	45 (41.7%)	20 (32.8%)	0.160
LCX	189 (26.3%)	13 (12.0%)	19 (31.1%)	
RCA	164 (22.8%)	29 (26.9%)	15 (24.6%)	
LM	19 (2.6%)	5 (4.6%)	2 (3.3%)	
Graft	19 (2.6%)	3 (2.8%)	1 (1.6%)	
Multiple	54 (7.5%)	13 (12.0%)	4 (6.6%)	

Significant *p* values are bold

Values are median [IQR] or *n* (%)

*CABG* indicates coronary artery bypass graft, *CAD* coronary artery disease, *ECG* electrocardiogram, *LAD* left anterior descending artery, *LCX*, left circumflex artery, *LM* left main coronary artery, *PCI* percutaneous coronary intervention, *RCA* right coronary artery

^*^Body mass index missing for 107 (12.0%) patients

### Independent predictors for the absence of chest discomfort in type 1 NSTEMI

**Fig. 2:**
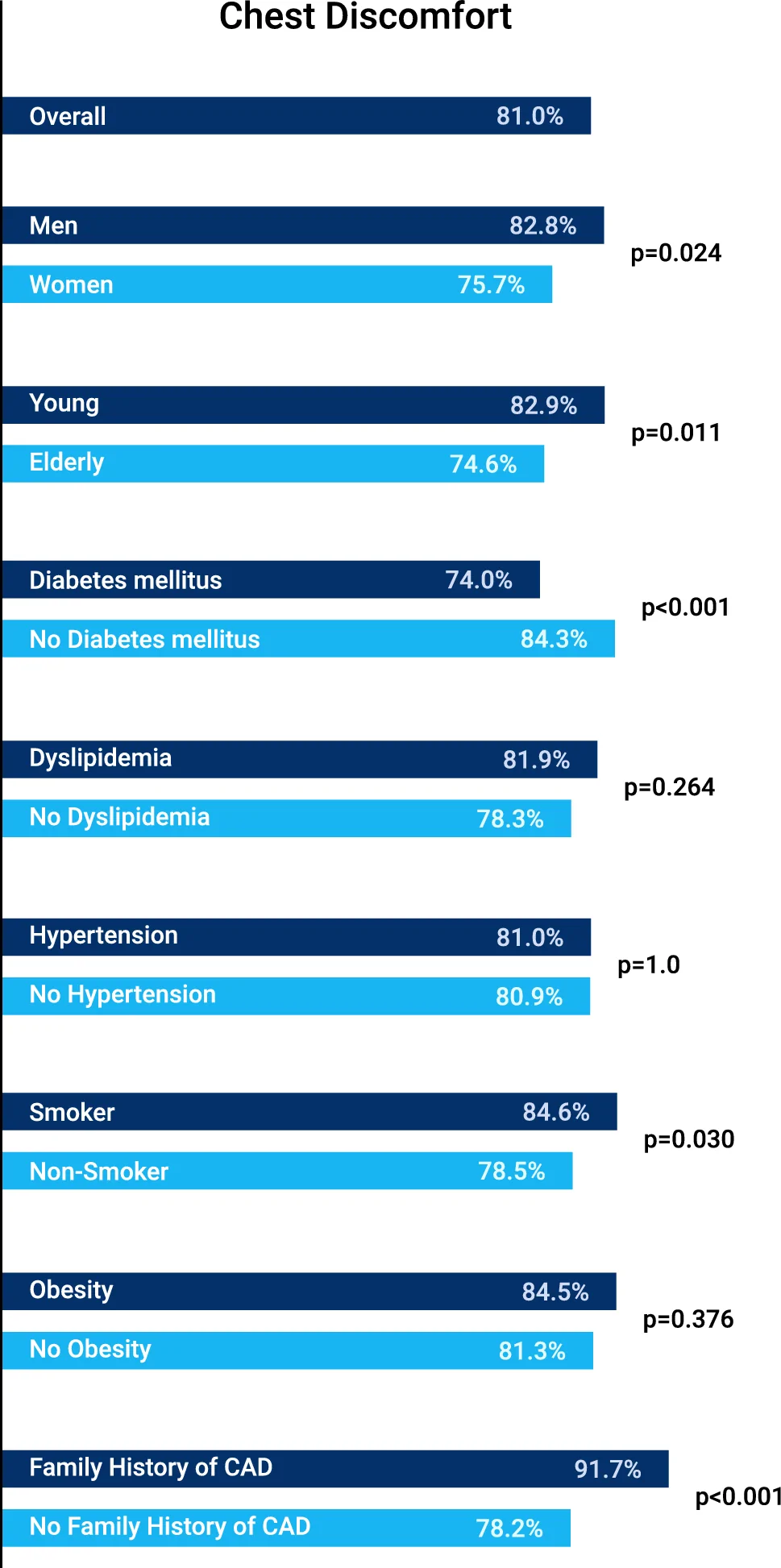
Differences in chest discomfort by demographics and cardiovascular risk factors. Overall rate of chest discomfort in type 1 NSTEMI and differences in chest discomfort by demographics and cardiovascular risk factors

Women with type 1 NSTEMI presented less often with chest discomfort than men (75.7 vs. 82.8%, *p* = 0.024, Fig. 2). Likewise, elderly patients presented less often with chest discomfort than their younger counterparts (74.6 vs. 82.9%, *p* = 0.011, Fig. 2). Sex and age differences regarding symptoms and patient characteristics are listed in Supplemental Table 1.

As compared to non-diabetic patients, patients with diabetes presented less often with chest discomfort (74.0 vs. 84.3%, *p* < 0.001, Fig. 2). No differences regarding the frequency of chest discomfort were observed between patients with and without dyslipidemia, obesity, and hypertension (Fig. 2). Smokers presented more often with chest discomfort than non-smokers (84.6 vs. 78.5%, *p* = 0.030, Fig. 2). Patients with a family history of premature CAD presented substantially more frequently with chest discomfort than those without a family history of CAD (91.7 vs. 78.2%, *p* < 0.001, Fig. 2). Furthermore, patients both with anemia (69.2 vs. 86.8%, *p* < 0.001) as well as atrial fibrillation or flutter at presentation (65.0 vs. 82.5%, *p* < 0.001) presented less frequently with chest discomfort than patients without. Differences in symptoms according to cardiovascular risk profile and comorbidities are detailed in Supplemental Table 2.

The location of the culprit lesion had no significant influence on the frequency of chest discomfort (*p* = 0.201, Supplemental Table 3). However, with increasing diameter stenosis of the culprit lesion, patients were more likely to report chest discomfort (*p* = 0.040, Supplemental Table 4).

For the variables listed above, older age (*p* < 0.001), female sex (*p* = 0.019), diabetes mellitus (*p* < 0.001), the absence of smoking (*p* = 0.025), the absence family history of premature CAD (*p* < 0.001), anemia (*p* < 0.001), atrial fibrillation or flutter at presentation (*p* < 0.001) and lower diameter stenoses of the culprit lesion (*p* = 0.030) correlated significantly with the absence of chest discomfort in an univariable regression analysis (Table 4). In a multivariable regression analysis, female sex (OR 1.517 [95% CI 1.025–2.229], *p* = 0.035), diabetes mellitus (OR 1.723 [95% CI 1.200–2.469], *p* = 0.003), the absence of a family history of premature CAD (OR 2.444 [95% CI 1.413–4.509], *p* = 0.002), anemia (OR 2.268 [95% CI 1.559–3.305], *p* < 0.001) and atrial fibrillation or flutter at presentation (OR 1.913 [95% CI 1.112–3.237], *p* = 0.017) independently predicted the absence of chest discomfort (Table 4).

**Table 4:** Univariable and multivariable regression analysis for predictors of absence of chest discomfort (*n* = 888 patients)

Variable	Univariable			Multivariable		
	OR	95% CI	P Value	OR	95% CI	*P* value
Age, years	1.031	1.016–1.046	** < 0.001**	1.005	0.988–1.023	0.543
Female	1.546	1.069–2.219	**0.019**	1.517	1.025–2.229	**0.035**
Diabetes mellitus	1.895	1.343–2.670	** < 0.001**	1.723	1.200–2.469	**0.003**
Dyslipidemia	0.795	0.551–1.159	0.225	–	–	–
Hypertension	0.996	0.646–1.577	0.987	–	–	–
Smoking	0.666	0.465–0.945	**0.025**	0.809	0.546–1.189	0.283
Obesity	0.800	0.507–1.229	0.321	–	–	–
No family history of CAD	3.082	1.820–5.596	** < 0.001**	2.444	1.413–4.509	**0.002**
Creatinine, mg/dl	1.002	0.925–1.051	0.929	–	–	–
Anemia	2.945	2.092–4.158	** < 0.001**	2.268	1.559–3.305	** < 0.001**
Atrial fibrillation/flutter	2.547	1.538–4.146	** < 0.001**	1.913	1.112–3.237	**0.017**
Culprit lesion				–	–	–
LAD	–	–	–			
LCX	0.714	0.445–1.125	0.153			
RCA	1.131	0.733–1.732	0.574			
LM	1.553	0.586–3.700	0.342			
Graft	0.887	0.251–2.457	0.833			
Multiple	1.327	0.706–2.399	0.362			
Diameter stenosis, %	0.982	0.966–0.999	**0.030**	0.983	0.967–1.001	0.062

Significant *p* values are bold

*CAD* indicates coronary artery disease, *CI* confidence interval, *LAD* left anterior descending artery, *LCX* left circumflex artery, *LM* left main coronary artery, *OR* odds ratio, *RCA* right coronary artery

### In-hospital outcomes

**Fig. 3:**
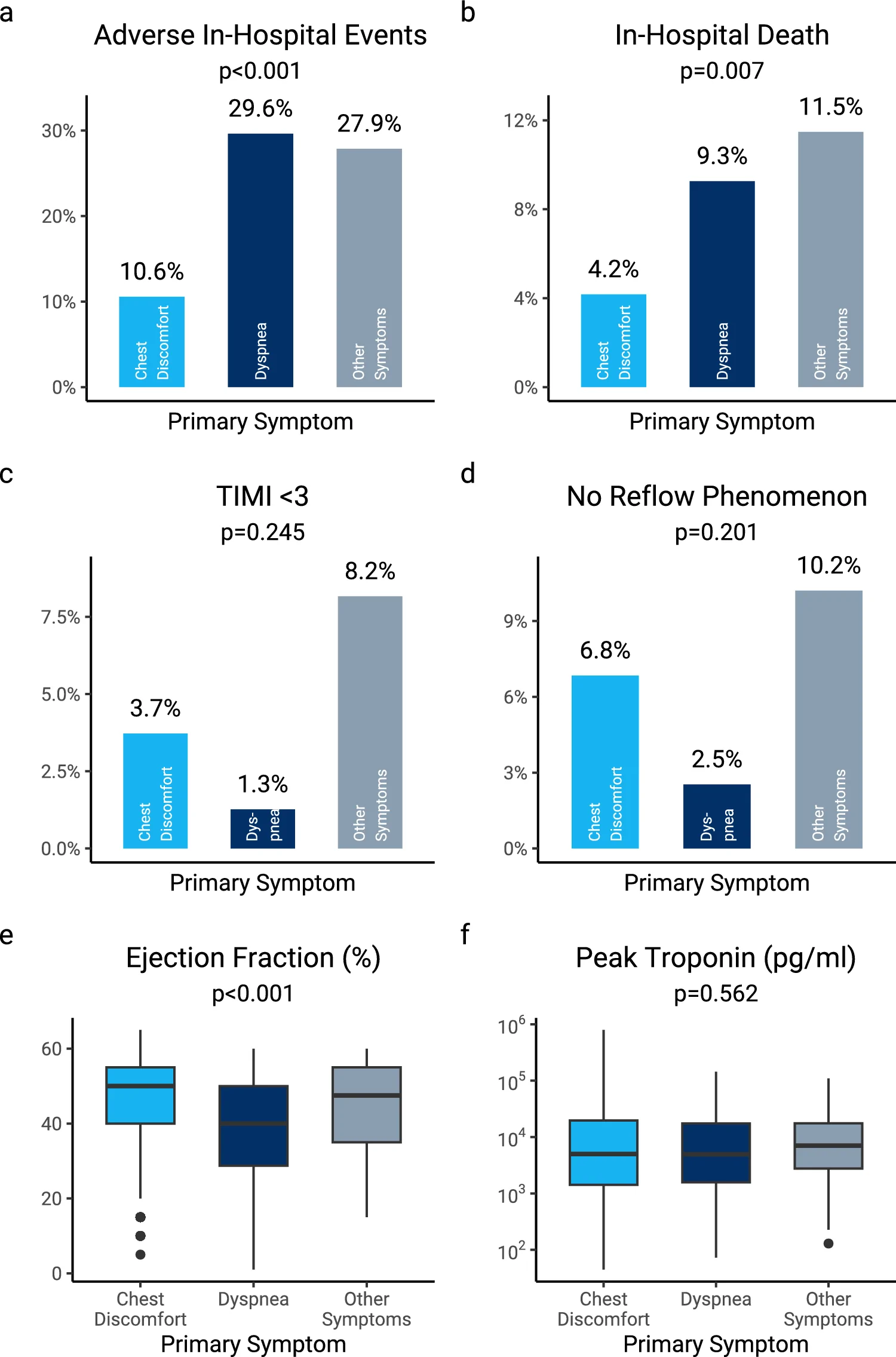
In-hospital outcomes. **a** Adverse in-hospital events, **b** in-hospital death, **c** TIMI flow after PCI, **d** no reflow phenomenon, **e** ejection fraction, and **f** peak troponin of patients with type 1 NSTEMI stratified by the primary symptom

The rate of adverse in-hospital events was higher for patients presenting with dyspnea (29.6%) or other symptoms (27.9%) as compared to those presenting with chest discomfort (10.6%, *p* < 0.001, Fig. 3a, Table 5). More specifically, the rates of in-hospital death (9.3% for dyspnea and 11.5% for other symptoms vs. 4.2% for chest discomfort, *p* = 0.007, Fig. 3b), cardiogenic shock (28.7% for dyspnea and 23.0% for other symptoms vs. 9.3% for chest discomfort, *p* < 0.001), and the need for mechanical ventilation (19.4% for dyspnea and 18.0% for other symptoms vs. 4.9% for chest discomfort, *p* < 0.001) were higher for patients presenting with other symptoms or dyspnea rather than chest discomfort.

**Table 5:** In-hospital outcomes by primary symptom (*n* = 888 patients)

	Chest discomfort (*n* = 719)	Dyspnea (*n* = 108)	Other symptoms (*n* = 61)	*P* value
Adverse in-hospital event	76 (10.6%)	32 (29.6%)	17 (27.9%)	** < 0.001**
In-hospital death	30 (4.2%)	10 (9.3%)	7 (11.5%)	**0.007**
Cardiogenic shock	67 (9.3%)	31 (28.7%)	14 (23.0%)	** < 0.001**
Mechanical ventilation	35 (4.9%)	21 (19.4%)	11 (18.0%)	** < 0.001**
Acute kidney injury*	72 (10.0%)	29 (26.9%)	9 (14.8%)	** < 0.001**
TIMI flow after PCI*				
TIMI 3	621 (96.3%)	78 (98.7%)	45 (91.8%)	0.245
TIMI 2	7 (1.1%)	0 (0%)	2 (4.1%)	
TIMI 1	7 (1.1%)	0 (0%)	0 (0%)	
TIMI 0	10 (1.6%)	1 (1.3%)	2 (4.1%)	
No reflow phenomenon*	44 (6.8%)	2 (2.5%)	5 (10.2%)	0.201
Ejection fraction*, %	50 [40–55]	40 [29–50]	48 [35–55]	** < 0.001**
Ischemic ECG changes	339 (47.1%)	46 (42.6%)	31 (50.8%)	0.549
Troponin, pg/ml	4990 [1420–19600]	4930 [1570–17400]	7030 [2770–17500]	0.562
Creatine kinase, U/l	355 [176–786]	393 [215–818]	453 [242–934]	0.102

Significant *p* values are bold

Values are median [IQR] or *n* (%)

*ECG* indicates electrocardiogram, *PCI* percutaneous coronary intervention, *TIMI* thrombolysis in myocardial infarction

^*^Ejection fraction was missing for 16 (1.8%) patients, and data for acute kidney injury for 23 (2.6%) patients. Information regarding TIMI flow after PCI was available for 773 (87.0%) patients and regarding no reflow phenomenon for 771 (86.8%) patients

The rate of acute kidney injury was highest for patients with dyspnea (26.9%) and lowest for patients with chest discomfort (10.0%, *p* < 0.001, Table 5). No significant differences regarding TIMI flow after PCI of the culprit lesion or occurrence of a no reflow phenomenon were observed (Fig. 3c, d, Table 5).

Left ventricular ejection fraction was lowest among patients with dyspnea (40% [IQR 29–50%]) and highest among patients with chest discomfort (50% [IQR 40–55%], *p* < 0.001, Fig. 3e, Table 5). No differences were observed regarding peak troponin or peak creatine kinase (Fig. 3f, Table 5).

### Timing of invasive management

The time from diagnosis to invasive management was longer for patients presenting with dyspnea (1410 min [IQR 368–4380 min]) or other symptoms (945 min [IQR 241–2210 min]) compared to patients presenting with chest discomfort (298 min [IQR 121–1060 min], *p* < 0.001).

**Fig. 4:**
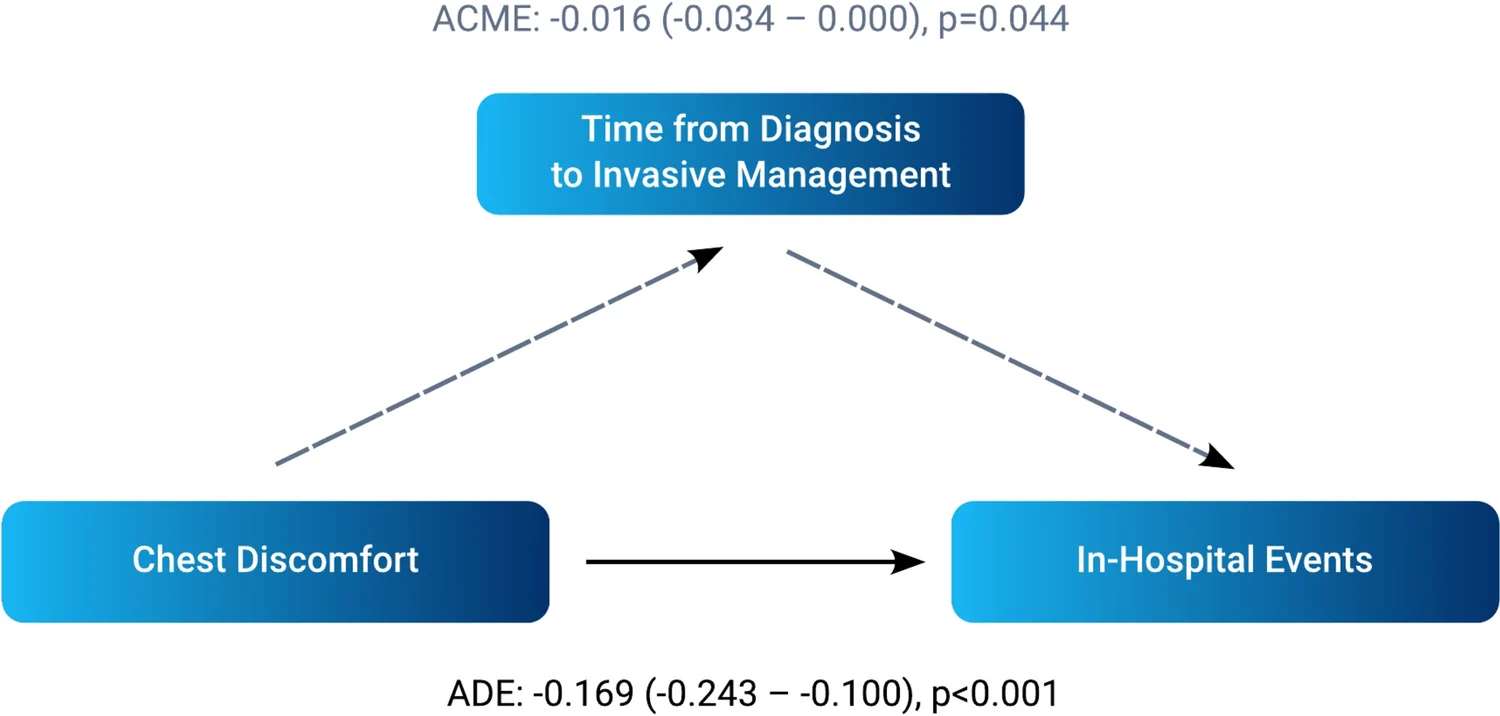
Indirect effect mediated by time from diagnosis to invasive management on in-hospital events. Causal mediation analysis for the direct effect of chest discomfort on in-hospital events and the indirect effect mediated by the time from diagnosis to invasive management among patients with type 1 NSTEMI. ACME indicates average causal mediation effect and ADE average direct effect

In the causal mediation analysis, both the total (*p* < 0.001) and direct (*p* < 0.001) effect of chest discomfort on adverse in-hospital events were highly significant. Additionally, the causal mediation effect of the time from diagnosis to invasive management for chest discomfort on adverse in-hospital events was significant (*p* = 0.044). Consequently, the higher rate of adverse in-hospital events of patients without chest discomfort did appear to be partially mediated by the observed longer times from diagnosis to invasive management with an average proportion mediated of 8.4% (95% CI −0.3–19.0%, *p* = 0.044). The conducted causal mediation analysis is illustrated in Fig. 4 and outlined in detail in Supplemental Table 5. Likewise, in a multivariable analysis for in-hospital events both absence of chest discomfort (*p* = 0.001) as well as the time from diagnosis to invasive management (*p* = 0.018) appeared to be one of the relevant predictors (Supplemental Table 6).

## Discussion

### Main findings

This study analyzed the spectrum of presenting symptoms in patients with NSTEMI focused on type 1 NSTEMI as well as the impact of the presenting symptoms on adverse in-hospital events in a large, single-center, all-comers, real-world registry. In line with previous findings [1, 8], roughly 80% of NSTEMI patients reported chest discomfort with or without accompanying dyspnea or other symptoms during presentation. Dyspnea without chest discomfort was present in about 10–15% of patients, while only other symptoms without chest discomfort or dyspnea were observed in the remaining 5–10%.

Furthermore, in patients with type 1 NSTEMI, female sex, diabetes mellitus, no family history of premature CAD, anemia, and atrial fibrillation or flutter at presentation were independent predictors for the absence of chest discomfort. The absence of chest discomfort was associated with worse in-hospital outcomes, which appeared to be partially mediated by the longer times from diagnosis to invasive management observed among these patients.

### Independent predictors for the absence of chest discomfort in type 1 NSTEMI

Chest discomfort is known to be less frequent in women compared to men presenting with acute myocardial infarction as lately shown in a meta-analysis including over 1 million patients [8]. However, this data did not allow a distinction to be made between type 1 and type 2 myocardial infarction. The authors therefore postulated in the discussion that these sex differences in symptom presentation might be explained by women having a type 2 infarction more often than men. Our results now provide the desired evidence that this sex difference also applies to the symptoms of patients with type 1 myocardial infarction. Accordingly, factors other than the type of myocardial infarction must be responsible for this. Plausible explanations include anatomical factors (smaller vessel diameters, lower stroke volume), differences in endocrine hormones (the role of estrogen in particular), differences in sympathetic and parasympathetic activity, and hematological differences (lower number of erythrocytes in women) [24]. However, despite sex differences regarding symptom presentation commonly addressed in literature and even the guideline [1], they do not appear to be as pronounced as often precepted given that female sex was the weakest independent predictor of all for the absence of chest discomfort.

Furthermore, diabetes mellitus was an independent predictor for the absence of chest discomfort in our registry. This verifies previous observations of diabetics being less likely to present with chest discomfort than non-diabetics [14, 25–27]. It is presumed diabetic neuropathy is the cause of the lower perception of chest discomfort [28].

Interestingly, the absence of a family history of premature CAD was another independent predictor for the absence of chest discomfort. A family history of CAD is known to be a risk factor for myocardial infarctions in patients presenting with chest pain [29]. Accordingly, it seems reasonable that patients with a family history of CAD are also more likely to show chest discomfort in case of myocardial infarction. A potential explanation could be that patients with a familiar disposition are known to be younger and that younger patients more often have chest discomfort as shown in various studies already [10, 13]. Even though age was not an independent predictor for chest discomfort in our study, there was a significant positive correlation between younger age and chest discomfort in the univariate analysis. Additionally, awareness of a family history of premature CAD might result in an improved interceptive sensibility for chest discomfort.

Furthermore, patients with atrial fibrillation or flutter at presentation as well as patients with anemia experienced chest discomfort less often in our registry. Due to the well-known fact that both atrial fibrillation and flutter as well as anemia can trigger a type 2 infarction due to increased oxygen consumption [30] and that patients with type 2 infarction are more likely to have atypical symptoms, patients with one of the two conditions and lack of chest discomfort are certainly at risk of a slowed invasive management in the presence of a type 1 myocardial infarction. In fact, in these patients, a type 2 myocardial infarction is often diagnosed merely based on clinical judgement and confirmatory invasive coronary angiography not performed [31]. However, our data show that it is precisely in these patients that special attention should be paid to additional factors not to miss type 1 myocardial infarction. In a recent prospective cohort study, systematic coronary angiography unveiled type 1 myocardial infarctions in about 5% of patients with a provisional diagnosis of type 2 myocardial infarction [32], which highlights the risk of not recognizing the need for revascularization in these patients. One explanation for the lack of typical symptoms in type 1 myocardial infarction could be the antianginal effect of the drugs especially the betablockers prescribed among these patients.

### In-hospital outcomes

The rate of adverse in-hospital events as well as in-hospital death was higher in the absence of chest discomfort in our study. Previous studies have already shown, that the absence of chest discomfort is associated with higher morbidity and an increased mortality [14, 25, 33]. Most interestingly, our study showed that the time from diagnosis to invasive management has an impact on patient outcome and is prolonged in patients without chest discomfort. Relevant delays in diagnosis and treatment among patients presenting without chest discomfort have been speculated to contribute to worse outcomes previously [33]. The latest ESC guidelines have already narrowed the time frames for invasive diagnostics in patients with NSTEMI and now recommend an invasive strategy immediately for patients at very high-risk and within 24 h for patients at high risk [1]. Besides cardiogenic shock, cardiac arrhythmias, electrocardiographic signs of ischemia, or mechanical complications, classification as very high risk is also possible only based on recurrent or ongoing chest pain refractory to medical treatment [1]. Given the results of this study, these classification criteria may be insufficient. Instead, in everyday clinical practice, particular attention should be paid to any—even atypical—persistent symptoms, which may reflect an acute coronary syndrome, especially in the patient groups shown to be at risk for atypical symptoms, so patients will be treated timely.

### Study limitations

This study presumably is the largest investigating symptoms of patients presenting with NSTEMI comprehensively. Nevertheless, this study only enrolled patients with confirmatory invasive coronary angiography to avoid the potential influence of incorrect clinical judgement on its results. Even though such an analysis for all patients presenting at chest pain units may be desirable, this would require systematic coronary imaging in these patients, which is currently not performed in clinical practice. Additionally, the underlying registry is only built upon the data of a single center and only included patients with a suspected NSTEMI but no other types of non-ST-elevation acute coronary syndromes like unstable angina. Symptoms were obtained by a physician as part of the history taking during the routine diagnostic workup and not systematically by a questionnaire. A thorough investigation of disparities in symptoms would also benefit from analyzing the influence of ethnicity. However, ethnicity has not been obtained in the registry, nor would this analysis have been feasible in a predominantly Caucasian population. Furthermore, often vague and sometimes even not remembered onset of symptoms—especially in patients without chest discomfort—made analysis on the time between symptom onset to diagnosis and treatment impossible, even though this would have been especially desirable regarding its impact on in-hospital outcomes. Finally, data was only collected until discharge and no follow-up was performed. Hence, regarding outcomes, only analysis of in-hospital outcomes was achievable.

## Conclusions

About 20% of patients with type 1 NSTEMI present without chest discomfort and about 1/3 of these do not even perceive dyspnea. Women, diabetics, patients without a family history of premature CAD, patients with anemia, and patients with atrial fibrillation or flutter at presentation reported chest discomfort less frequently. Since there was a correlation between the absence of chest discomfort and worse in-hospital outcomes, which could be partly explained due to delayed invasive management, special attention should be paid to any atypical symptoms and especially to the named patient groups in everyday life.

## Supplementary Information

Below is the link to the electronic supplementary material.

Supplementary file1 (DOCX 40 KB)

## Data Availability

The data that support the findings of this study are available on request from the corresponding author. The data are not publicly available due to privacy or ethical restrictions.
