# Supplementary material for: Absence of chest discomfort in type 1 NSTEMI patients: predictors and impact on outcome
Source: Clin Res Cardiol. 2025 Mar 13;115(10):1723–34. doi: 10.1007/s00392-025-02628-1 (PMC13562127; doi:10.1007/s00392-025-02628-1)
Supplement: Supplementary file 1 [file 392_2025_2628_MOESM1_ESM.docx]

# Supplemental Material

## Supplemental Table 1 Differences in Symptoms and Patient Characteristics by Sex and Age (*n=*888 patients)

|  | Male  (*n=*662) | Female  (*n=*226) | P Value |
| --- | --- | --- | --- |
| Chest discomfort | 548 (82.8%) | 171 (75.7%) | **0.024** |
| Dyspnea | 297 (43.4%) | 100 (44.2%) | 0.876 |
| Other symptoms  Non-thoracic pain  Orthostatic  Gastrointestinal  Diaphoresis  Vegetative  Fatigue  Neurologic | 249 (37.6%)  52/249 (20.9%)  60/249 (24.1%)  41/249 (16.5%)  53/249 (21.3%)  37/249 (14.9%)  34/249 (13.7%)  18/249 (7.2%) | 97 (42.9%)  20/97 (20.6%)  26/97 (26.8%)  31/97 (32.0%)  13/97 (13.4%)  20/97 (20.6%)  9/97 (9.3%)  7/97 (7.2%) | 0.182  1.0  0.700  **0.002**  0.127  0.256  0.354  1.0 |
| Primary symptom  Chest discomfort  Dyspnea  Other symptoms | 548 (82.8%)  77 (11.6%)  37 (5.6%) | 171 (75.7%)  31 (13.7%)  24 (10.6%) | **0.024**  0.478  **0.015** |
| Age, years | 67 [58-78] | 74 [66-81] | **<0.001** |
| Obesity* | 137 (20.7%) | 56 (24.8%) | 0.313 |
| Hypertension | 537 (81.1%) | 199 (88.1%) | **0.022** |
| Diabetes mellitus | 213 (32.2%) | 75 (33.2%) | 0.843 |
| Dyslipidemia | 486 (73.4%) | 172 (76.1%) | 0.478 |
| Smoking | 287 (43.4%) | 70 (31.0%) | **0.001** |
| Family history of CAD | 136 (20.5%) | 45 (19.9%) | 0.914 |
| Previous PCI | 224 (33.8%) | 60 (26.5%) | 0.052 |
| Previous CABG | 60 (9.1%) | 18 (8.0%) | 0.713 |
| Creatinine, mg/dl | 1.02 [0.86-1.29] | 0.86 [0.71-1.08] | **<0.001** |
| Hemoglobin, mg/dl | 14.1 [12.2-15.3] | 12.8 [11.3-13.8] | **<0.001** |
| Troponin, pg/ml | 6100 [1610-21300] | 3590 [1130-13000] | **0.004** |
| Creatine Kinase, U/l | 408 [199-881] | 296 [149-645] | **<0.001** |
| Hearth Rhythm on ECG  Sinus Rhythm  Atrial fibrillation/flutter  Other | 603 (91.1%)  57 (8.6%)  2 (0.3%) | 203 (89.8%)  23 (10.2%)  0 (0%) | 0.557 |
| Ischemic ECG Changes | 288 (43.5%) | 128 (56.6%) | **<0.001** |
| Ejection Fraction*, % | 45 [40-55] | 50 [40 - 55] | 0.421 |
| Culprit lesion  LAD  LCX  RCA  LM  Graft  Multiple | 245 (37.0%)  164 (24.8%)  154 (23.3%)  20 (3.0%)  20 (3.0%)  59 (8.9%) | 94 (41.6%)  57 (25.2%)  54 (23.9%)  6 (2.7%)  3 (1.3%)  12 (5.3%) | 0.342 |
|  | **Young**  **(*n=*683)** | **Elderly**  **(*n=*205)** | **P Value** |
| Chest discomfort | 566 (82.9%) | 153 (74.6%) | **0.011** |
| Dyspnea | 275 (40.3%) | 112 (54.6%) | **<0.001** |
| Other symptoms  Non-thoracic pain  Orthostatic  Gastrointestinal  Diaphoresis  Vegetative  Fatigue  Neurologic | 273 (40.0%)  57/273 (20.9%)  62/273 (22.7%)  56/273 (20.5%)  60/273 (22.0%)  40/273 (14.7%)  33/273 (12.1%)  22/273 (8.1%) | 73 (35.6%)  15/73 (20.5%)  24/73 (32.9%)  16/73 (21.9%)  6/73 (8.2%)  17/73 (23.3%)  10/73 (13.7%)  3/73 (4.1%) | 0.298  1.0  0.103  0.920  **0.013**  0.112  0.864  0.366 |
| Primary symptom  Chest discomfort  Dyspnea  Other symptoms | 566 (82.9%)  69 (10.1%)  48 (7.0%) | 153 (74.6%)  39 (19.0%)  13 (6.3%) | **0.011**  **<0.001**  0.855 |
| Sex, male | 524 (76.7%) | 138 (67.3%) | **0.009** |
| Obesity* | 163 (23.9%) | 30 (14.6%) | **0.010** |
| Hypertension | 544 (79.6%) | 192 (93.7%) | **<0.001** |
| Diabetes mellitus | 218 (31.9%) | 70 (34.1%) | 0.608 |
| Dyslipidemia | 501 (73.4%) | 157 (76.6%)) | 0.403 |
| Smoking | 320 (46.9%) | 37 (18.0%) | **<0.001** |
| Family history of CAD | 153 (22.4%) | 28 (13.7%) | **0.009** |
| Previous PCI | 199 (29.1%) | 85 (41.5%) | **0.001** |
| Previous CABG | 47 (6.9%) | 31 (15.1%) | **<0.001** |
| Creatinine, mg/dl | 0.96 [0.81-1.17] | 1.12 [0.88-1.44] | **<0.001** |
| Hemoglobin, mg/dl | 14.1 [12.4-15.2] | 12.4 [10.9-13.7] | **<0.001** |
| Troponin, pg/ml | 4640 [1310-18800] | 6190 [2150-19700] | 0.066 |
| Creatine Kinase, U/l | 373 [177-845] | 356 [212-735] | 0.934 |
| Hearth Rhythm on ECG  Sinus Rhythm  Atrial fibrillation/flutter  Other | 646 (94.6%)  35 (5.1%)  2 (0.3%) | 160 (78.0%)  45 (22.0%)  0 (0%) | **<0.001** |
| Ischemic ECG Changes | 320 (46.9%) | 96 (46.8%) | 1.0 |
| Ejection Fraction*, % | 50 [40-55] | 45 [35 - 55] | **0.004** |
| Culprit lesion  LAD  LCX  RCA  LM  Graft  Multiple | 259 (37.9%)  183 (26.8%)  163 (23.9%)  17 (2.5%)  9 (1.3%)  52 (7.6%) | 80 (39.0%)  38 (18.5%)  45 (22.0%)  9 (4.4%)  14 (6.8%)  19 (9.3%) | **<0.001** |

* Body mass index missing for 107 (12.0%) patients and ejection fraction for 16 (1.8%) patients.

Values are median [IQR] or n (%). CABG indicates coronary artery bypass graft; CAD, coronary artery disease; ECG, electrocardiogram; LAD, left anterior descending artery; LCX, left circumflex artery; LM, left main coronary artery; PCI, percutaneous coronary intervention; RCA, right coronary artery.

## Supplemental Table 2 Differences in Symptoms by Cardiovascular Risk Factors and Comorbidities (*n=*888 patients)

|  | Diabetes Mellitus  (*n=*288) | No Diabetes Mellitus  (*n=*600) | P Value |
| --- | --- | --- | --- |
| Chest discomfort | 213 (74.0%) | 506 (84.3%) | **<0.001** |
| Dyspnea | 147 (53.8%) | 232 (38.7%) | **<0.001** |
| Other symptoms  Non-thoracic pain  Orthostatic  Gastrointestinal  Diaphoresis  Vegetative  Fatigue  Neurologic | 111 (38.5%)  31/111 (27.9%)  26/111 (23.4%)  25/111 (22.5%)  20/111 (18.0%)  15/111 (13.5%)  17/111 (15.3%)  8/111 (7.2%) | 235 (39.2%)  41/235 (17.4%)  60/235 (25.5%)  47/235 (20.0%)  46/235 (19.6%)  42/235 (17.9%)  26/235 (11.1%)  17/235 (7.2%) | 0.916  **0.036**  0.772  0.691  0.844  0.387  0.345  1.0 |
| Primary symptom  Chest discomfort  Dyspnea  Other symptoms | 213 (74.0%)  52 (18.1%)  23 (8.0%) | 506 (84.3%)  56 (9.3%)  38 (6.3%) | **<0.001**  **<0.001**  0.441 |
|  | **Dyslipidemia**  **(*n=*658)** | **No Dyslipidemia**  **(*n=*223)** | **P Value** |
| Chest discomfort | 539 (81.9%) | 180 (78.3%) | 0.264 |
| Dyspnea | 296 (45.0%) | 91 (39.6%) | 0.177 |
| Other symptoms  Non-thoracic pain  Orthostatic  Gastrointestinal  Diaphoresis  Vegetative  Fatigue  Neurologic | 254 (38.6%)  39/254 (17.0%)  61/254 (26.5%)  53/254 (23.0%)  47/254 (20.4%)  44/254 (19.1%)  22/254 (9.6%)  14/254 (6.1%) | 92 (40.0%)  13/92 (14.9%)  19/92 (21.8%)  18/92 (20.7%)  17/92 (19.5%)  10/92 (11.5%)  15/92 (17.2%)  11/92 (12.6%) | 0.767  0.793  0.477  0.766  0.984  0.148  0.086  0.089 |
| Primary symptom  Chest discomfort  Dyspnea  Other symptoms | 539 (81.9%)  79 (12.0%)  40 (6.1%) | 180 (78.3%)  29 (12.6%)  21 (9.1%) | 0.264  0.902  0.155 |
|  | **Hypertension**  **(*n=*736)** | **No Hypertension**  **(*n=*152)** | **P Value** |
| Chest discomfort | 596 (81.0%) | 123 (80.9%) | 1.0 |
| Dyspnea | 335 (45.5%) | 52 (34.2%) | **0.014** |
| Other symptoms  Non-thoracic pain  Orthostatic  Gastrointestinal  Diaphoresis  Vegetative  Fatigue  Neurologic | 271 (36.8%)  57/271 (21.0%)  69/271 (25.5%)  56/271 (20.7%)  47/271 (17.3%)  47/271 (17.3%)  31/271 (11.4%)  23/271 (8.5%) | 75 (49.3%)  15/75 (20.0%)  17/75 (22.7%)  16/75 (21.3%)  19/75 (25.3%)  10/75 (13.3%)  12/75 (16.0%)  2/75 (2.7%) | **0.005**  0.973  0.730  1.0  0.164  0.514  0.389  0.141 |
| Primary symptom  Chest discomfort  Dyspnea  Other symptoms | 596 (81.0%)  94 (12.8%)  46 (6.3%) | 123 (80.9%)  14 (9.2%)  15 (9.9%) | 1.0  0.153  0.156 |
|  | **Smoker**  **(*n=*357)** | **Non-Smoker**  **(*n=*531)** | **P Value** |
| Chest discomfort | 302 (84.6%) | 417 (78.5%) | **0.030** |
| Dyspnea | 156 (43.6%) | 231 (43.5%) | 1.0 |
| Other symptoms  Non-thoracic pain  Orthostatic  Gastrointestinal  Diaphoresis  Vegetative  Fatigue  Neurologic | 130 (36.4%)  36/130 (27.7%)  38/130 (29.2%)  27/130 (20.8%)  27/130 (20.8%)  14/130 (10.8%)  12/130 (9.2%)  8/130 (6.2%) | 216 (40.7%)  36/216 (16.7%)  48/216 (22.2%)  45/216 (20.8%)  39/216 (18.1%)  43/216 (19.9%)  31/216 (14.4%)  17/216 (7.9%) | 0.227  **0.021**  0.183  1.0  0.631  **0.039**  0.219  0.702 |
| Primary symptom  Chest discomfort  Dyspnea  Other symptoms | 302 (84.6%)  39 (10.9%)  16 (4.5%) | 417 (78.5%)  69 (13.0%)  45 (8.5%) | **0.030**  0.412  **0.030** |
|  | **Obesity***  **(*n=*193)** | **No Obesity***  **(*n=*588)** | **P Value** |
| Chest discomfort | 163 (84.5%) | 478 (81.3%) | 0.376 |
| Dyspnea | 80 (41.5%) | 252 (42.9%) | 0.796 |
| Other symptoms  Non-thoracic pain  Orthostatic  Gastrointestinal  Diaphoresis  Vegetative  Fatigue  Neurologic | 70 (36.3%)  17/70 (24.3%)  16/70 (22.9%)  14/70 (20.0%)  22/70 (31.4%)  9/70 (12.9%)  7/70 (10.0%)  5/70 (7.1%) | 220 (37.4%)  37/220 (16.8%)  54/220 (24.5%)  46/220 (20.9%)  39/220 (17.7%)  41/220 (18.6%)  29/220 (13.2%)  18/220 (8.2%) | 0.842  0.222  0.899  1.0  **0.023**  0.351  0.621  0.979 |
| Primary symptom  Chest discomfort  Dyspnea  Other symptoms | 163 (84.5%)  19 (9.8%)  11 (5.7%) | 478 (81.3%)  73 (12.4%)  37 (6.3%) | 0.376  0.405  0.901 |
|  | **Family History of CAD**  **(*n=*181)** | **No Family History of CAD**  **(*n=*707)** | **P Value** |
| Chest discomfort | 166 (91.7%) | 553 (78.2%) | **<0.001** |
| Dyspnea | 66 (36.5%) | 321 (45.4%) | **0.038** |
| Other symptoms  Non-thoracic pain  Orthostatic  Gastrointestinal  Diaphoresis  Vegetative  Fatigue  Neurologic | 71 (39.2%)  16/71 (22.5%)  16/71 (22.5%)  16/71 (22.5%)  15/71 (21.1%)  12/71 (16.9%)  7/71 (9.9%)  5/71 (7.0%) | 275 (38.9%)  56/275 (20.4%)  70/275 (25.5%)  56/275 (20.4%)  51/275 (18.5%)  45/275 (16.4%)  36/275 (13.1%)  20/275 (7.3%) | 1.0  0.812  0.724  0.812  0.746  1.0  0.593  1.0 |
| Primary symptom  Chest discomfort  Dyspnea  Other symptoms | 166 (91.7%)  8 (4.4%)  7 (3.9%) | 553 (78.2%)  100 (14.1%)  54 (7.6%) | **<0.001**  **<0.001**  0.104 |
|  | **Anemia**  **(*n=*295** | **No Anemia**  **(*n=*593)** | **P Value** |
| Chest discomfort | 204 (69.2%) | 515 (86.8%) | **<0.001** |
| Dyspnea | 172 (58.3%) | 215 (36.3%) | **<0.001** |
| Other symptoms  Non-thoracic pain  Orthostatic  Gastrointestinal  Diaphoresis  Vegetative  Fatigue  Neurologic | 107 (36.3%)  15/107 (14.0%)  27/107 (25.2%)  24/107 (22.4%)  10/107 (9.3%)  19/107 (17.8%)  15/107 (14.0%)  15/107 (14.0%) | 239 (40.3%)  57/239 (23.8%)  59/239 (24.7%)  48/239 (20.1%)  56/239 (23.4%)  38/239 (15.9%)  28/239 (11.7%)  10/239 (4.2%) | 0.277  0.053  1.0  0.724  **0.003**  0.784  0.672  **0.002** |
| Primary symptom  Chest discomfort  Dyspnea  Other symptoms | 204 (69.2%)  67 (22.7%)  24 (8.1%) | 515 (86.8%)  41 (6.9%)  37 (6.2%) | **<0.001**  **<0.001**  0.362 |
|  | **Atrial Fibrillation or Flutter**  **(*n=*80)** | **No Atrial Fibrillation or Flutter**  **(*n=*808)** | **P Value** |
| Chest discomfort | 52 (65.0%) | 667 (82.5%) | **<0.001** |
| Dyspnea | 43 (53.8%) | 344 (42.6%) | 0.071 |
| Other symptoms  Non-thoracic pain  Orthostatic  Gastrointestinal  Diaphoresis  Vegetative  Fatigue  Neurologic | 35 (43.8%)  8 (22.9%)  13 (37.1%)  7 (20.0%)  3 (8.6%)  10 (28.6%)  7 (20.0%)  3 (8.6%) | 311 (38.5%)  64 (20.6%)  73 (23.5%)  65 (20.9%)  63 (20.3%)  47 (15.1%)  36 (11.6%)  22 (7.1%) | 0.424  0.924  0.117  1.0  0.149  0.073  0.245  1.0 |
| Primary symptom  Chest discomfort  Dyspnea  Other symptoms | 52 (65.0%)  18 (22.5%)  10 (12.5%) | 667 (82.5%)  90 (11.1%)  51 (6.3%) | **<0.001**  **0.005**  0.064 |

* 131 (11.9%) patients excluded due to missing body mass index.

Values are n (%). CVD indicates coronary artery disease.

## Supplemental Table 3 Differences in Symptoms by Culprit Lesion (*n=*794 patients)

|  | LM  (*n=*26) | LAD  (*n=*339) | LCX  (*n=*221) | RCA  (*n=*208) | P Value |
| --- | --- | --- | --- | --- | --- |
| Chest discomfort | 19 (73.1%) | 274 (80.8%) | 189 (85.5%) | 164 (78.8%) | 0.201 |
| Dyspnea | 18 (69.2%) | 153 (45.1%) | 84 (38.0%) | 85 (40.9%) | **0.014** |
| Other symptoms  Non-thoracic pain  Orthostatic  Gastrointestinal  Diaphoresis  Vegetative  Fatigue  Neurologic | 10 (38.5%)  1/10 (10.0%)  4/10 (40.0%)  1/10 (10.0%)  1/10 (10.0%)  2/10 (20.0%)  3/10 (30.0%)  1/10 (10.0%) | 131 (38.6%)  20/131 (15.3%)  34/131 (26.0%)  29/131 (22.1%)  25/131 (19.1%)  17/131 (13.0%)  20/131 (15.3%)  10/131 (7.6%) | 83 (37.6%)  19/83 (22.9%)  17/83 (20.5%)  16/83 (19.3%)  15/83 (18.1%)  16/83 (19.3%)  8/83 (9.6%)  11/83 (13.3%) | 85 (40.9%)  21/85 (24.7%)  22/85 (25.9%)  17/85 (20.0%)  19/85 (22.4%)  11/85 (12.9%)  10/85 (11.8%)  3/85 (3.5%) | 0.915  0.251  0.531  0.805  0.767  0.552  0.261  0.142 |
| Primary symptom  Chest discomfort  Dyspnea  Other symptoms | 19 (73.1%)  5 (19.2%)  2 (7.7%) | 274 (80.8%)  45 (13.3%)  20 (5.9%) | 189 (85.5%)  13 (5.9%)  19 (8.6%) | 164 (78.8%)  29 (13.9%)  15 (7.2%) | 0.201  **0.014**  0.679 |

Values are n (%). LAD indicates left anterior descending artery; LCX, left circumflex artery; LM, left main coronary artery; RCA, right coronary artery.

## Supplemental Table 4 Differences in Symptoms by Diameter Stenosis (*n=*888 patients)

|  | 40-49%  (*n=*3) | 50-59%  (*n=*3) | 60-69%  (*n=*15) | 70-79%  (*n=*28) | 80-89%  (*n=*44) | 90-99%  (*n=*578) | 100%  (*n=*217) | P Value |
| --- | --- | --- | --- | --- | --- | --- | --- | --- |
| Chest discomfort | 2 (66.7%) | 3 (100%) | 10 (66.7%) | 26 (92.9%) | 30 (68.2%) | 463 (80.1%) | 185 (85.3%) | **0.040** |
| Dyspnea | 1 (33.3%) | 1 (33.3%) | 6 (40.0%) | 8 (28.6%) | 21 (47.7%) | 258 (44.6%) | 92 (42.4%) | 0.731 |
| Other symptoms  Non-thoracic pain  Orthostatic  Gastrointestinal  Diaphoresis  Vegetative  Fatigue  Neurologic | 2 (66.7%)  1/2 (50.0%)  1/2 (50.0%)  0/2 (0%)  0/2 (0%)  0/2 (0%)  0/2 (0%)  0/2 (0%) | 0 (0%)  —  —  —  —  —  —  — | 8 (53.3%)  1/8 (12.5%)  2/8 (25.0%)  2/8 (25.0%)  1/8 (12.5%)  2/8 (25.0%)  0/8 (0%)  0/8 (0%) | 11 (39.3%)  0/11 (0%)  3/11 (27.3%)  4/11 (36.4%)  3/11 (27.3%)  5/11 (45.5%)  2/11 (18.2%)  0/11 (0%) | 16 (36.4%)  1/16 (6.3%)  2/16 (12.5%)  6/16 (37.5%)  2/16 (12.5%)  2/16 (12.5%)  5/16 (31.3%)  1/16 (6.3%) | 202 (34.9%)  45/202 (22.3%)  52/202 (25.7%)  32/202 (15.8%)  34/202 (16.8%)  32/202 (15.8%)  27/202 (13.4%)  17/202 (8.4%) | 107 (49.3%)  24/107 (22.4%)  26/107 (24.3%)  28/107 (26.2%)  26/107 (24.3%)  16/107 (15.0%)  9/107 (8.4%)  7/107 (6.5%) | **0.006**  0.240  0.832  0.081  0.525  0.159  0.121  0.827 |
| Primary symptom  Chest discomfort  Dyspnea  Other symptoms | 2 (66.7%)  1 (33.3%)  0 (0%) | 3 (100%)  0 (0%)  0 (0%) | 10 (66.7%)  3 (20.0%)  2 (13.3%) | 26 (92.9%)  1 (3.6%)  1 (3.6%) | 30 (68.2%)  11 (25.0%)  3 (6.8%) | 463 (80.1%)  68 (11.8%)  47 (8.1%) | 185 (85.3%)  24 (11.1%)  8 (3.7%) | **0.040**  0.072  0.342 |

Values are n (%).

## Supplemental Table 5 Causal Mediation Analysis for Direct Effect of Chest Discomfort on In-Hospital Events and Indirect Effect Mediated by Time from Diagnosis to Invasive Management (*n=*888 patients)

|  | Estimate | 95% Confidence Interval | P Value |
| --- | --- | --- | --- |
| ACME (no chest discomfort) | -0.021 | -0.044 – 0.000 | **0.044** |
| ACME (chest discomfort) | -0.011 | -0.023 – 0.000 | **0.044** |
| ACME (average) | -0.016 | -0.034 – 0.000 | **0.044** |
| ADE (no chest discomfort) | -0.174 | -0.250 – -0.100 | **<0.001** |
| ADE (chest discomfort) | -0.164 | -0.241 – -0.090 | **<0.001** |
| ADE (average) | -0.169 | -0.243 – -0.100 | **<0.001** |
| Total effect | -0.185 | -0.258 – -0.110 | **<0.001** |
| Proportion mediated (no chest discomfort) | 0.113 | -0.004 – 0.250 | **0.044** |
| Proportion mediated (chest discomfort) | 0.055 | -0.002 – 0.140 | **0.044** |
| Proportion mediated (average) | 0.084 | -0.003 – 0.190 | **0.044** |

ACME indicates average causal mediation effect and ADE average direct effect.

## Supplemental Table 6 Multivariable Regression Analysis for In-hospital Events in Type 1 NSTEMI (*n=*888 patients)

| Characteristic | OR | 95% Confidence Interval | P Value |
| --- | --- | --- | --- |
| Age, years | 1.030 | 1.003 - 1.059 | **0.030** |
| Female | 1.253 | 0.640 - 2.389 | 0.500 |
| Absence of chest discomfort | 3.296 | 1.624 - 6.581 | **0.001** |
| Creatinine, mg/dl | 0.988 | 0.772 - 1.056 | 0.860 |
| Hemoglobin, mg/dl | 1.707 | 0.910 - 3.191 | 0.093 |
| Maximum Creatine Kinase, U/l | 1.000 | 1.000 - 1.000 | **0.004** |
| Ischemic ECG changes | 1.496 | 0.850 - 2.666 | 0.165 |
| Ejection fraction, % | 0.957 | 0.935 - 0.980 | **<0.001** |
| Diagnosis-to-Puncture time, min | 1.000 | 1.000 - 1.000 | **0.018** |
| Culprit lesion  LAD  LCX  RCA  LM  Graft  Multiple | —  0.713  0.511  0.820  1.582  0.895 | —  0.338 - 1.449  0.209 - 1.150  0.111 - 3.689  0.297 - 6.479  0.290 - 2.428 | —  0.300  0.112  0.817  0.397  0.832 |
| Diameter stenosis, % | 1.002 | 0.968 - 1.043 | 0.925 |

ECG indicates electrocardiogram; LAD, left anterior descending artery; LCX, left circumflex artery; LM, left main coronary artery; OR, odds ratio; RCA, right coronary artery.
